# Barriers to gene exchange in hybridizing field crickets: the role of male courtship effort and cuticular hydrocarbons

**DOI:** 10.1186/1471-2148-14-65

**Published:** 2014-03-28

**Authors:** Luana S Maroja, Zachary M McKenzie, Elizabeth Hart, Joy Jing, Erica L Larson, David P Richardson

**Affiliations:** 1 Department of Biology, Williams College, 31 Morley Drive, 01267 Williamstown, MA, USA; 2 Department of Mathematics and Statistics, Williams College, 01267 Williamstown, MA, USA; 3 Division of Biological Sciences, University of Montana, 59802 Missoula, MT, USA; 4 Department of Chemistry, Williams College, 01267 Williamstown, MA, USA

**Keywords:** *Gryllus firmus*, *Gryllus pennsylvanicus*, Behavior, Barrier to gene exchange, Pre-zygotic, Pheromone

## Abstract

**Background:**

Pre-zygotic barriers often involve some form of sexual selection, usually interpreted as female choice, as females are typically the choosier sex. However, males typically show some mate preferences, which are increasingly reported. Here we document previously uncharacterized male courtship behavior (effort and song) and cuticular hydrocarbon (CHC) profiles in the hybridizing crickets *Gryllus firmus* and *G. pennsylvanicus*. These two species exhibit multiple barriers to gene exchange that act throughout their life history, including a behavioral barrier that results in increased time to mate in heterospecific pairs.

**Results:**

We demonstrated that male mate choice (as courtship effort allocation) plays a more important role in the prezygotic behavioral barrier than previously recognized. In gryllids females ultimately decide whether or not to mate, yet we found males were selective by regulating courtship effort intensity toward the preferred (conspecific) females. Females were also selective by mating with more intensely courting males, which happened to be conspecifics. We report no differences in courtship song between the two species and suggest that the mechanism that allows males to act differentially towards conspecific and heterospecific females is the cuticular hydrocarbon (CHC) composition. CHC profiles differed between males and females of both species, and there were clear differences in CHC composition between female *G. firmus* and *G. pennsylvanicus* but not between the males of each species.

**Conclusion:**

Although many barriers to gene exchange are known in this system, the mechanism behind the mate recognition leading to reduced heterospecific mating remains unknown. The CHC profiles might be the phenotypic cue that allow males to identify conspecifics and thus to adjust their courtship intensity accordingly, leading to differential mating between species.

## Background

Pre-zygotic barriers to gene exchange can play a large role in reducing gene flow between species; these barriers act earlier in the life cycle and have the potential to restrict hybridization more than later acting barriers [1]. Many of these barriers involve some form of sexual selection, usually interpreted as female choice, as females typically invest the most in the offspring [2] and thus tend to be choosier. However, male mate choice has now been described even in species where males do not invest in offspring care [3-6]. Furthermore, it has been theoretically demonstrated that male and mutual mate choice can evolve in a wide range of circumstances [7-9], especially when females are encountered simultaneously (rather than sequentially), when there is variability in female fertility [3] or when females prefer males that court intensively [10].

The hybridizing field crickets - *Gryllus firmus*[11] and *Gryllus pennsylvanicus*[12] - form an extensive hybrid zone [13-16] and have multiple barriers to gene exchange [17-21] including an early acting pre-mating behavioral barrier [22]. Conspecific pairs mate faster than heterospecific pairs [22] and, although male courtship behavior has never been analyzed, the time to mate barrier has been interpreted as female choice. In gryllid crickets females must ultimately mount the male and cooperate in the transfer of the spermatophore, making forced copulation impossible and leaving the ultimate mating decision to the female. Here we test if males are able to regulate courtship intensity depending on whether they encounter a conspecific or heterospecific female.

To understand the speciation process, we need to understand not only the barriers to gene exchange, but also the mechanisms that allow individuals to act differentially towards conspecifics and heterospecifics. Although barriers to gene exchange between *G. firmus* and *G. pennsylvanicus* have been well documented, the mechanism behind mate recognition remains elusive (here we avoided the misleading term “species recognition” [23], using “mate recognition” for within as well as between species mate choice). In most species, recognition mechanisms involve appearance (i.e. morphology) or behavior (e.g. courtship), however the very closely related *G. firmus* and *G. pennsylvanicus* are morphologically and behaviorally similar [16,22]. Pheromones are another common communication mechanism providing reliable long- or short-distance communication. In particular, the non-volatile cuticular hydrocarbons (CHC) play an important role in mate recognition both within and between species in a wide range of insect taxa [24-30], including other crickets [31-33]. CHCs serve as contact pheromones and might be the primary mechanism used by species that lack obvious morphological or behavioral differences [27]. Therefore we also investigate if there are differences in CHC profiles between species that could explain the pre-mating behavioral barrier to gene exchange.

Our results suggest that although females ultimately decide whether or not to mate, males regulate courtship intensity and male choice may play a more important role in the interspecific pre-mating behavioral barrier than previously recognized. Furthermore we report sex specificity in the CHC profiles in both species, and clear differences in CHC composition between female *G. firmus* and *G. pennsylvanicus* but not between males of each species. We hypothesize that males may use the CHC profiles as a means to identify conspecifics and adjust their courtship intensity accordingly, leading to differential mating between species.

## Methods

### Collection

In August 2010 and 2011, we collected late instar *G. firmus* (GF) nymphs from Point Judith, RI (41°22′; −71°29′) and Guilford, CT (41°.13′, -72°40′) and *G. pennsylvanicus* (GP) nymphs in Pownal, VT (42°45′; −73°13′) and Ithaca, NY (42°25′, −76°.29′), all allopatric pure species populations. We sorted nymph crickets by sex and species and raised them at room temperature (25°C) in plastic cages (35 × 31 × 13 cm) with ad libitum food (50/50 mixture of Purina Cat Chow® and LM Bonanza Rabbit Food®), cotton-plugged water vials, and egg cartons for shelter. We separated newly molted adults every 2–3 days.

### Mating trials

For the mating trials, we only used females from Pownal, VT (*G. pennsylvanicus*) and Pt. Judith, RI (*G. firmus*). Seven- to eight-day old virgin females were haphazardly assigned to one of two treatments. We placed the female in a mating chamber (100 × 25 mm petri dish lined with moist paper) with either a conspecific or heterospecific male. All males had been adults between 7 and 10 days. For each mating pair we recorded the start of courtship behavior, the time to mating, and male and female pronotal width measured to the nearest 0.1 mm. If there was no spermatophore transfer after 60 min, the cross was recorded as failed and the female was immediately placed with another male of the same species. If the second male also failed to mate after 60 min, the cross was recorded as an unsuccessful mating.

### Courtship and mating failure data

Proportion of failed courtship (male did not start courtship) and failed mating (no successful mating resulted) data were analyzed using generalized linear models (GLMs) implemented in R. 3.0.1 [34]. The vector of successes and failures was the dependent variable while the independent variables were male species, female species. To avoid using the same female more than once in the analyses, we only considered the 1^st^ male a female was exposed to. We fitted our data to GLM with binomial errors and logit link [35]. Visual inspection of error structure indicated a good fit to the model.

### Time to mate data

Only individuals that successfully mated were analyzed for time to mate. Data were analyzed using generalized linear models (GLMs) implemented in R. 3.0.1 [34]. Because this data refers to the time to an event (mating), we fitted it to Gamma errors with inverse link as recommended for survival analysis [35]. Visual inspection of error structure indicated a good fit to the model. The dependent variable was time to mate and independent variables were time to call, male order (1^st^ male the female was exposed to or 2^nd^ male in cases where the 1^st^ male failed to mate), male species and female species and size (females were not duplicated in the analyses, since they were only exposed to a second male if the first failed to mate). We subsequently simplified the model removing variables that were not significant.

### Chemical analyses

For the chemical analysis, we used crickets from all four populations but not the same individuals that were used in the mating trials. Individuals were between 7–15 days old and were kept in same species/sex boxes of 10 individuals each. To avoid plastic contamination and minimize individual to individual contamination, 5–7 days prior to extraction each cricket was individually housed in a glass container.

Because females are larger than males, we extracted cuticular hydrocarbons by placing female crickets in 3 dram glass vials containing 3 mL of HPLC-grade hexane and male crickets in 2 dram glass vials containing 2 mL of HPLC-grade hexane for five to seven minutes [36]. The solution was then filtered with PallLife Sciences Acrodisc (13 mm 0.2 μ nylon membrane) syringe filters to remove particulates, and analyzed with an Agilent Technologies 7890A GC System with an AT 190915–433 30 m × 25 μm × 0.25 μm column attached to an AT 5975C inert XL EI/CI MSD with Triple-Axis Detector MS System, obtaining chromatograms and both EI and CI mass spectra. For the GC method we used a 1 μL or 2 μL injection with an injection temperature of 250°C. The column was held at an initial temperature of 60°C for 4 min followed by a 10°C/min increase to 180°C and then a 3°C/min increase to the final temperature of 260°C, which was then held for 10 min (helium as a carrier gas). All samples were run in duplicate to ensure precision of the GC-MS instrument. Integration parameters were the following: initial area reject = 0; peak width = 0.027; shoulder detection = off; threshold = 14.

To analyze the GC-MS data, we scored a total of 17 peaks representing all seven typical male peaks and most of the female peaks excluding only two peaks that were difficult to score in some individuals (i.e. were a small plateau instead of a peak). To score the peaks, we used the percent of the total area contributed by each peak and then scaled the scored peaks to add up to 100% in each individual. All individuals were scored for all of the 17 peaks; although males rarely exhibited female peaks, females typically exhibited both male and female peaks. These data were analyzed with principal component analysis as performed by the ‘prcomp’ function in “stats” package implemented in R. 3.0.1 [34]. Statistical significance of species, sex and population were assessed by an ANOVA permutation test (10,000 permutations) with the “Vegan” package [37]. After confirming a difference between sexes, we analyzed males and females separately.

### Phonotaxis

For the phonotaxis analysis we also used males from all four populations. Because we were interested in courtship song (used when the female is on sight and the only song our experimental females were exposed to) and not calling song (long distance song to attract females), we placed a male and female cricket of same species in a petri dish lined with moist paper and with holes drilled into the lid. We then placed the petri dish in a chamber (35 × 31 × 13 cm) with a microphone attached to the side. This chamber was then enclosed in a 75 × 50 × 60 cm recording chamber with a constant temperature of 25°C, the outer chamber, originally designed for recording bird songs, was constructed of Lucite and lined on four sides with acoustic foam [38]. The crickets were recorded using an Audio-Technica 8010 condenser microphone, high-pass filtered and amplified (cutoff of 500 Hz), and digitized (16 bit, 44 kHz) using SoundEdit16 (Macromedia). We collected 10-min recordings of the courtship songs of each individual, which were then cut to 10-second clips to highlight areas of high courtship intensity. We measured eight variables from each clip using SoundEdit16: chirp duration, chirp period, pulses per chirp, pulse duration, pulse period, chirp peak frequency, pulse peak frequency, and number of harmonics per chirp. These data were analyzed in the same way as the CHC data (above). Statistical significance of species, population and male age were assessed by an ANOVA permutation test (10,000 permutations).

## Results

### Courtship and mating failure

**Figure 1:**
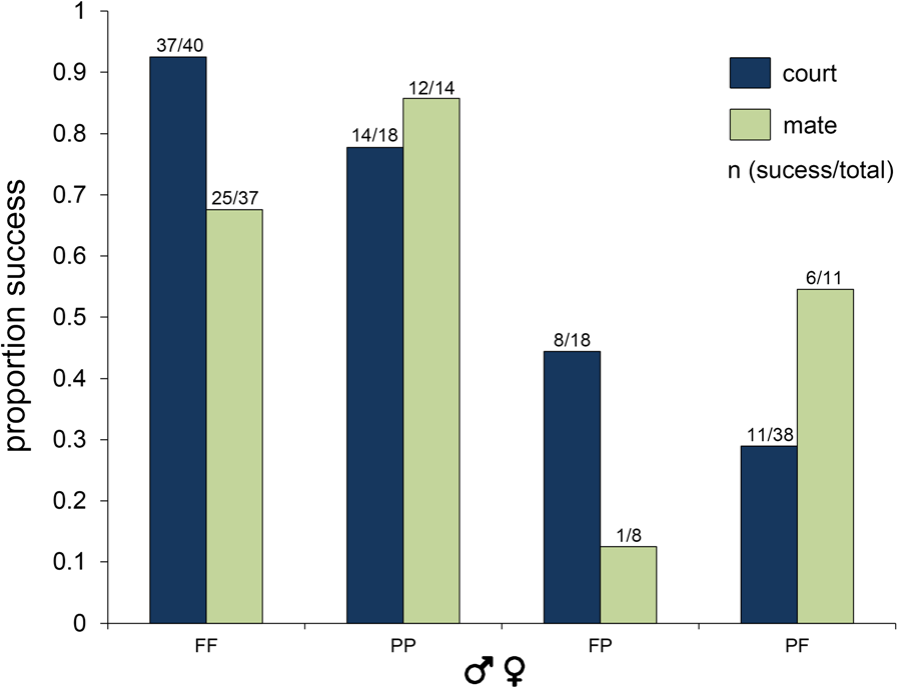
**Courtship and mating success.** Proportion of individuals that succeeded in courting (blue) or mating (green) considering only 1^st^ males. Since courtship is required for mating, only males that courted were analyzed for mating proportion. Numbers above bar represent the number of crosses (successful/total). First letter denotes male species and second letter denotes female species (F for *G. firmus* and P for *G. pennsylvanicus*).

Both male species (χ^2^ = 13.52, df = 5, *P* < 0.0002) and male–female species interaction (χ^2^ = 27.87, df = 4, *P* < 0.0001) were significant in determining the proportion of failed courtship (44 out of 114 trials, Figure 1), female species was not significant. Male *G. firmus* and males paired to conspecific females were more likely to initiate courtship (GF♂:GF♀: 92.5%, GP♂:GP♀: 77.8%, GF♂:GP♀ 44.4%, GP♂:GF♀: 28.9% courtship success).

Considering only 1^st^ males that initiated courtship (n = 70), the proportion of mating failure (n = 26, Figure 1) was influenced only by the interaction between males and females (χ^2^ = 10.62, df = 4, *P* < 0.001) and, as expected, conspecifics were more likely to mate (GF♂:GF♀: 67.6%, GP♂:GP♀: 85.7%, GF♂:GP♀: 12.5%, GP♂:GF♀: 54.5% mating success).

### Time to mate

**Figure 2:**
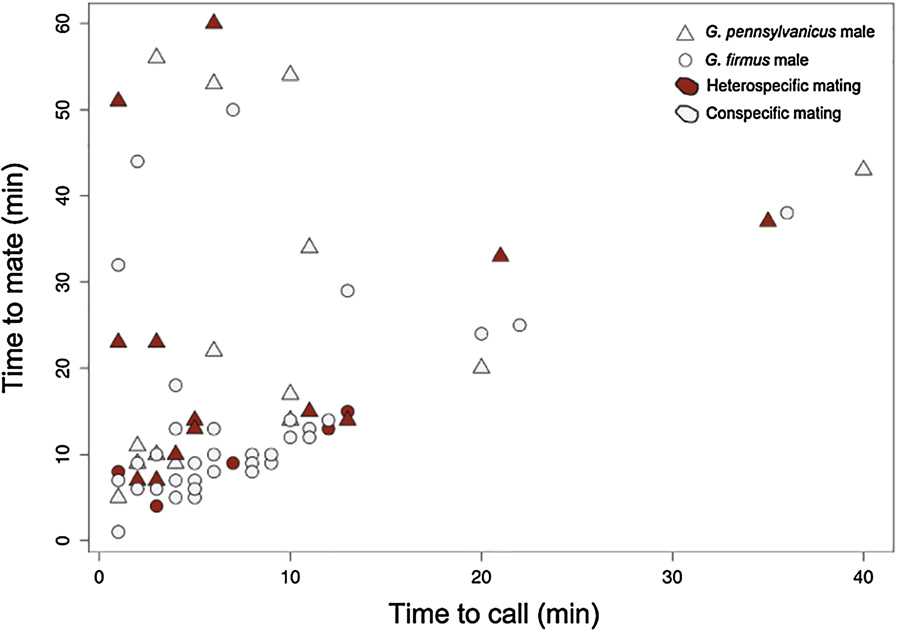
**Time to mate in relation to time to first call.** Results show only successful matings (n = 73), time to first call refers to the start of courtship behavior. Triangles represent *G. pennsylvanicus* males and circles represent *G. firmus* males. Conspecific matings are shown in light blue and heterospecific matings are shown in red. Time to mate was influenced by time to first call (*P = 0.01;* no mating took place in the absence of calling). There were no differences in time to mate between conspecific and heterospecific crosses however male *G. firmus* were faster to mate than male *G. pennsylvanicus* (*P = 0.006*).

Considering only the 73 (two crosses excluded due to missing data) out of 184 crosses that resulted in successful mating (nGF♂:GF♀: 38; nGP♂:GP♀: 15; nGF♂:GP♀: 5; nGP♂:GF♀: 13), time to mate (Figure 2) was influenced by the time to first call (*F*_*1,*69_ = 6.12, *P* = 0.01) and male species (*F*_*1,*67_ = 7.63, *P* = 0.006), *G. firmus* males mated faster (13.2 min for GF and 24.2 min for GP) and were also more vigorous during courtship (courting louder, continuously, and aggressively walking backward towards the female). Female species and male–female interaction (conspecific or heterospecific) did not influence time to mate (*F*_*1,*66_ = 0.09 and *F*_*1,*60_ = 0.07, P > 0.05 respectively).

### Cuticular hydrocarbon analysis

**Figure 3:**
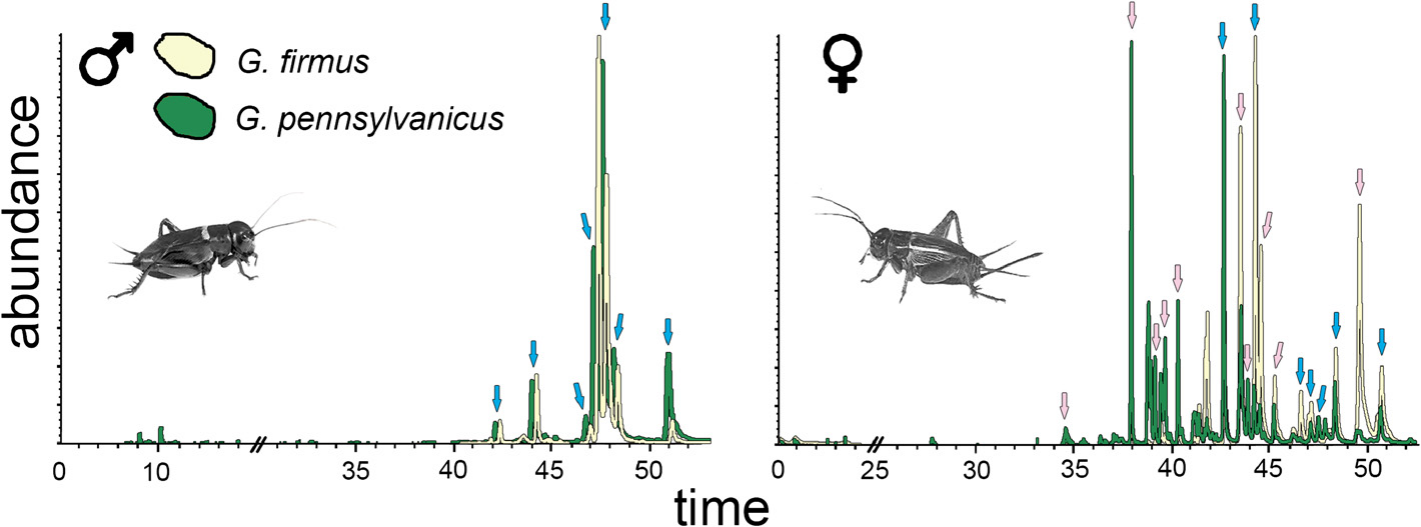
**Overlaid chromatograms of the two species.***G. firmus* CHC profile is shown in yellow and *G. pennsylvanicus* is shown in green. Males (left) and females (right). The X-axis is the retention time in minutes and the Y-axis is the relative CHC peak abundance. Arrows represent the scored peaks, typical male peaks (blue, n = 7) and female peaks (pink, n = 10), all peaks (when present) were measured in all individuals. Individuals used in this figure are shown in Additional file 1: Figure S1.

To analyze the gas chromatography results, we scored 17 peaks (Figure 3, Table 1) in 138 individuals (nGP♂ = 26, nGP♀ = 28; nGF♂ = 41, nGF♀ = 43). Of these peaks, seven were present in all males and some of the females (“male peaks”) and ten were present in most females but rarely in males (“female peaks”), if a peak was not present in an individual we recorded the peak abundance as zero. The recorded peaks (Table 1) had the following mean retention time (and standard deviation) in minutes (M for “male peaks” and F for “female peaks”): F1, 34.79 (0.01); F2, 38.14 (0.02); F3, 39.35 (0.01); F4, 39.88 (0.01); F5, 40.52 (0.02); M1, 42.86 (0.02); F6, 43.67 (0.03); F7,44.12 (0.02); M2, 44.43 (0.02); F8, 44.70 (0.03); F9, 45.42 (0.03); M3, 47.39 (0.04); M4, 47.78 (0.04); M5, 48.09 (0.04); M6, 48.55 (0.04); F10, 49.68 (0.03) and M7, 50.87 (0.04) minutes (Figure 3, Table 1).

**Table 1:** Average percent area and standard deviation (parenthesis) for scored peak in each group

**Peaks**	**GP** ♀ **(n = 28)**		**GF** ♀ **(n = 43)**		**GP** ♂ **(n = 26)**		**GF** ♂ **(n = 41)**	
	**Mean (SD)**	**% zero**	**Mean (SD)**	**% zero**	**Mean (SD)**	**% zero**	**Mean (SD)**	**% zero**
F1	1.6 (2.1)	28.6	3.8 (6.1)	11.6	3.5 (4.1)	3.8	0.6 (0.8)	7.3
F2	13.3 (16.9)	14.3	3.6 (5.4)	20.9	0 (0)	100	0 (0)	100
F3	1.2 (2.2)	67.9	0.2 (0.5)	86.0	0 (0)	100	0 (0)	100
F4	2.6 (5.0)	50.0	1.5 (3.0)	69.8	0 (0)	100	0 (0)	100
F5	3.5 (4.4)	32.1	4.0 (5.8)	20.9	0 (0)	100	0 (0)	100
M1	15.6 (9.3)	0.0	13.2 (8.3)	4.7	4.8 (3.3)	0	3.7 (2.0)	0
F6	11.1 (13.0)	28.6	15.2 (11.8)	7.0	0.3 (0.8)	84.6	0.1 (0.7)	87.8
F7	2.8 (4.6)	57.1	0.8 (3.4)	69.8	0.3 (1.6)	96.2	0 (0.02)	97.6
M2	8.9 (7.7)	0	16.4 (9.3)	0	9.7 (5.4)	0	9.0 (3.1)	0
F8	2.9 (3.4)	35.7	3.9 (3.9)	20.9	0 (0)	92.3	0.1 (0.4)	75.6
F9	1.5 (1.7)	21.4	2.1 (1.9)	16.3	0.3 (0.4)	50	0.2 (0.3)	61
M3	1.7 (2.0)	35.7	1.2 (2.1)	41.9	4.8 (4.8)	0	3.8 (2.4)	0
M4	10.9 (12.2)	21.4	4.4 (10.3)	60.5	22.3 (10.5)	0	37.9 (7.6)	0
M5	10.9 (13.1)	14.3	4.6 (9.2)	48.8	32.7 (10.5)	0	32.9 (4.3)	0
M6	6.6 (5.6)	7.1	7.9 (6.0)	2.3	14.0 (5.2)	0	8.0 (2.6)	0
F10	1.7 (4.5)	64.3	4.3 (5.7)	14.0	0 (0)	100	0 (0)	100
M7	3.1 (3.3)	17.9	12.9 (17.1)	2.3	7.4 (4.2)	0	3.7 (3.1)	0

The “% zero” refers to percentage of individuals lacking a particular peak. The seven typical male peaks (present in all males and many females) are designated “M” and 10 female peaks (rarely present in males) are designated “F”. All peaks were scored in all individuals.

**Figure 4:**
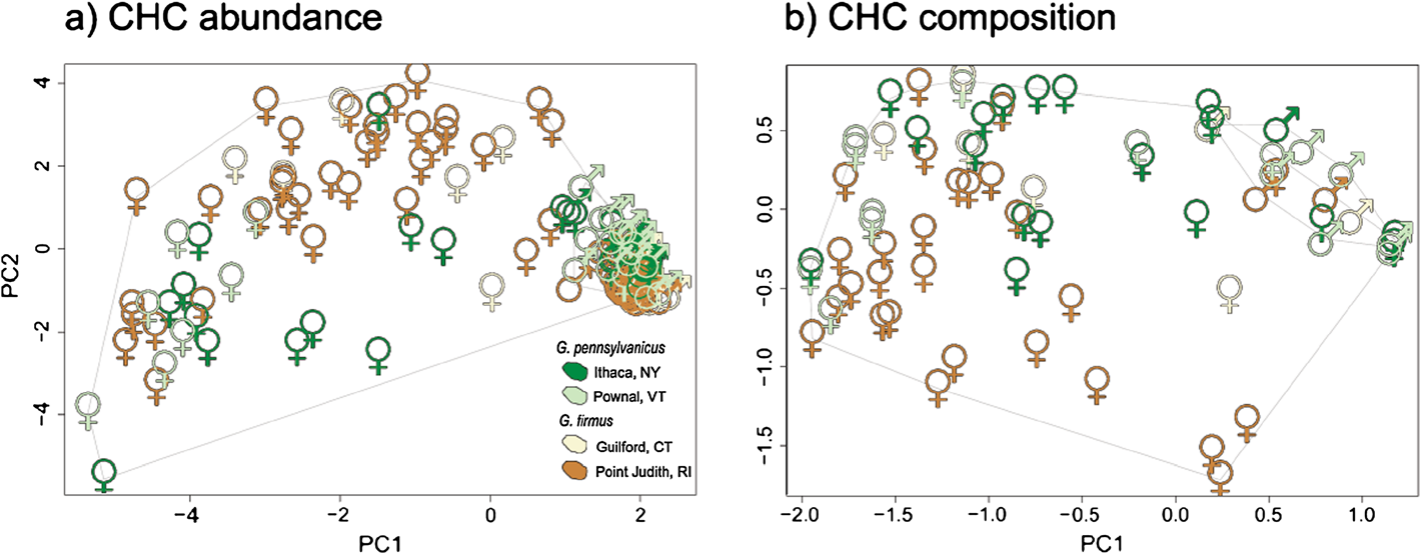
**Principal component analysis of CHC profiles. a)** CHC abundance and **b)** CHC composition (each male point represents multiple individuals of identical composition of both species). Colors represent populations: *G. firmus,* the beach cricket, is shown in sand colors yellow (Guilford, CT) and orange (Pt Judith, RI) while the inland field cricket, *G. pennsylvanicus,* is shown in green (Ithaca, NY) and light green (Pownal, VT). For CHC abundance there are significant differences between sexes and species for both males and females. For CHC composition (presence absence of peaks), only females had significant differences between species (males had similar composition, Figure 3).

Principal component analysis of CHC peak abundances (Figure 4 and Additional file 1: Figure S1) showed significant differences between sexes (GP *F*_1,50_ = 290.64, P < 0.0001; GF *F*_1,80_ = 139.31, *P* < 0.0001) and species (females *F*_1,67_ = 90.51, *P* = 0.0002; males *F*_1,63_ = 24.6, *P* = 0.0001) but not between populations (males *F*_2,63_ = 2.12, *P* = 0.062; females *F*_2,67_ = 1.24, *P* = 0.25). In contrast, the analysis of CHC composition (peak presence/absence), showed no differences between *G. firmus* and *G. pennsylvanicus* males (species *F*_1,63_ = 1.06, *P* = 0.35; populations *F*_2,63_ = 1.1, *P* = 0.35, Figure 3), but significant differences in female CHC composition both between species and populations (species *F*_1,68_ = 5.2, *P* = 0.0001; population *F*_2,68_ = 2.5, *P* = 0.0026).

Males usually exhibited all seven peaks (M1-M7) but occasionally a male would have a very low concentration of female peaks F1, F6, F7, F8 and F9 (Table 1, Figure 3). Females had a more complex and variable CHC profile (Table 1) with up to 19 peaks (two of which were not scored) including all typical male peaks.

**Figure 5:**
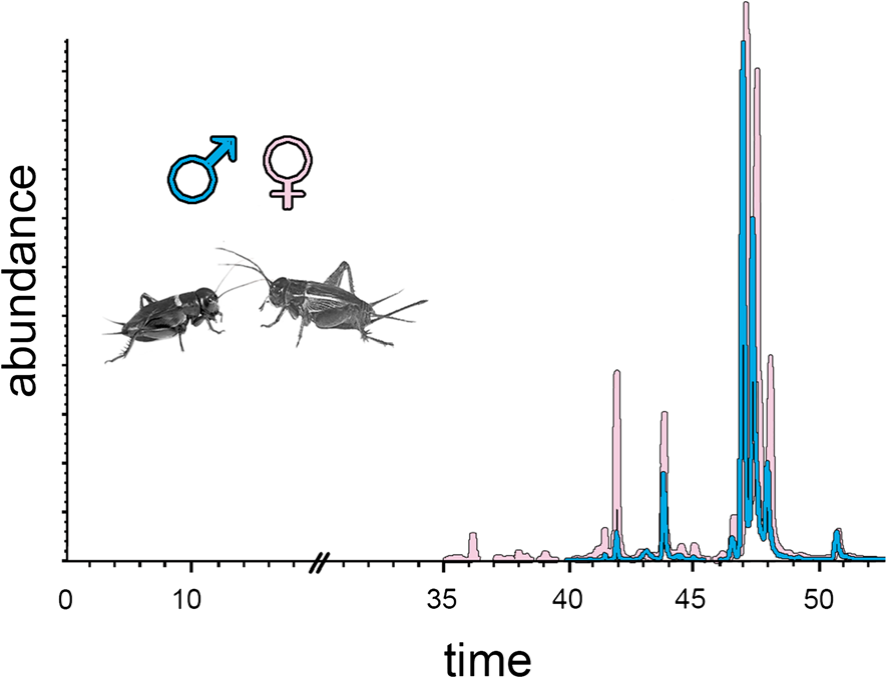
**Overlaid chromatograms of a “male like” female *G. firmus *(pink) and male *G. firmus *(blue).** The X-axis is the retention time in minutes and the Y-axis is the relative CHC peak abundance. Individuals used in this figure are shown in Additional file 1: Figure S1.

In both species, some females had a CHC profile almost identical to the typical male profile (Figure 5 and Additional file 1: Figure S1), with the exception of a higher prevalence of female peaks at very low frequencies in some individuals (typically below 1%). These “male likes” comprised 32.1% (9 out of 28) of the *G. pennsylvanicus* females and 11.6% (5 out of 43, with only 2 fully inside the male distribution) of the *G. firmus* females, a nearly statistically significant difference between species (exact Fisher test, *P* = 0.06).

To roughly categorize compounds present in the cuticular hydrocarbon mixture, we analyzed commercially available alkanes and alcohol standard mixtures. The retention times of the peaks in these mixtures did not match any of the profile peaks, but suggest that any alkanes present in the cricket hydrocarbon profile contain 23–34 carbons, indicating that the compounds present are not particularly volatile.

### Phonotaxis

We quantified courtship song characteristics in 16 *G. pennsylvanicus* and 11 *G. firmus* males. From the power spectra analysis, energy of both species’ songs is concentrated in the range between 2 kHz and 16 kHz, with spectral maxima always between 4 and 5 kHz.

The three most variable measurements were chirp period, pulse duration, and pulses per chirp. Although there was a high degree of variation among the individuals (CV ranging from 21% to 58% with a similar distribution between the two species), PC analysis of the eight courtship song variables showed no significant differences between species (*F*_1,20_ = 0.75, *P* = 0.41), populations (*F*_2,20_ = 0.19, *P* = 0.89) or an effect of male age (*F*_1,20_ = 0.09, *P* = 0.86). Pulse rate (the inverse of pulse period), which was suggested to be a driving factor in female preferences in *Laupala cerasina* and *L. eukolea* crickets [39], was more variable in *G. firmus* than *G. pennsylvanicus* (F- test, *F*_15,10_ = 6.03, P < 0.05) but did not show differences in magnitude between the two species (Wilcoxon Rank Sum test *U*_11,16_ = 123, *P* > 0.05).

## Discussion

The present study reports previously uncharacterized male courtship behavior and cuticular hydrocarbon profiles in the hybridizing crickets *Gryllus firmus* and *G. pennsylvanicus*. These two species exhibit multiple barriers to gene exchange that act throughout their life history, including a behavioral barrier that results in increased time to mate in heterospecific pairs (Maroja et al. [22]). This reluctance to mate with heterospecifics has previously been attributed to a female mate choice. Here, we demonstrated that male mate choice (as courtship effort allocation) plays a more important role in the interspecific prezygotic behavioral barrier than previously recognized. Furthermore cuticular hydrocarbon composition, which differs between females of the two species but not between males, might be the mechanism underlying male mate choice.

### Male courtship effort influences mating

Male courtship effort was an important factor in mating outcome (Figures 1 and 2). Our results show that males often fail to court heterospecific females (there was more courtship in conspecific pairs). When courtship intensity is sufficient to elicit a female response (i.e. successful mating), the female species did not influence time to mate (there was no significant effect of female species or male–female interaction on time to mate). This suggests that females will mate to any male that is vigorously courting. Heterospecific pairs are also more likely to fail to mate (Figure 1). Either females favor conspecific males for reasons unrelated to courtship effort or heterospecific males court less vigorously and thus fall below the threshold to elicit a female response [40].

In conspecific crosses, *Gryllus firmus* males typically courted vigorously and continuously throughout the mating trial. In contrast, *G. pennsylvanicus* males courted less vigorously (softly and did not walk aggressively towards the female) and often with an extensive time between first courtship effort and subsequent calling bouts (LSM and JJ personal observation). This difference in courtship intensity and aggression could be a result of differences in population densities; *G. firmus* often have higher population densities and less spaced populations than *G. pennsylvanicus* (e.g. [14]). Increased population densities have been shown to lead to agonistic behavior in field crickets, when populations densities are manipulated [41] or change naturally over the season [42]. Crickets in this study were exposed to constant densities in the lab (10–15 conspecifics of same sex), but it is possible that *G. firmus* has evolved agonistic behaviors as result of differences in natural populations. This could lead to more male-male competition (e.g. [43]) and increased courtship effort by *G. firmus* males. This difference in behavior could explain why females of both species mated faster with the vigorously courting *G. firmus*. It is also important to notice that heterospecific courtship effort from a *G. pennsylvanicus* male represents a clear case of misdirected courtship [44] as *G. firmus* females are unable to produce hybrid offspring [17]. Heterospecific courtship is thus more wasteful for a *G. pennsylvanicus* male than *G. firmus* male. Our results suggest that the prezygotic behavioral barrier (time to mate), originally interpreted as female choice [22], may be strongly influenced by male courtship effort. Indeed, results pointing towards faster time to mate in conspecific pairs were observed only in *G. firmus* females (and male courtship effort was not quantified [22]), which could suggest asymmetrical female preference (as observed in grasshoppers [45]) or could be interpreted as a result of the vigorous courtship behavior of *G. firmus* males in relation to the heterospecific *G. pennsylvanicus* males. The faster time to mate in conspecific *G. firmus* pairs can thus be explained by differential courtship effort between species, and not only by a preference for conspecifics by *G. firmus* females.

Courtship behavior is a costly signal (e.g. [46-48]) and it is thought to act as an honest signal of male quality [49,50]; males that court less vigorously might represent low quality individuals [3,40]. As a result it can be difficult to differentiate male choice through adjustments in courtship intensity from female choice based on male courtship vigor. The fact that we observed males differentially courting conspecific and heterospecific females, suggests that males adjust courtship effort to particular female types and that this is not simply a reflection of male quality. It is interesting that the very tendency of females to select males by courtship effort (well documented gryllids [51-54]) might lead to the evolution of male choosiness even in species where males do not invest in offspring. South et al. [10] modeled a system in which male choosiness evolved when females preferred males that courted with added intensity. Males that invested a larger proportion of their total courtship effort towards preferred females tended to acquire matings, thereby increasing the frequency of male “choosiness” alleles. In this scenario female choice plays a direct role on the evolution of male choice. Indeed, in many species males have been shown to be selective, as demonstrated in the allocation of sperm in *Gryllus*[55] and allocation of courtship effort in many other animal species [6,56-59]. Here males were selective by adjusting their courtship effort toward the preferred (conspecific) females, and females were selective by mating with more intensely courting males, which happened to (usually) be conspecifics.

### No differences in courtship song

While the barriers to gene exchange in this system have been well documented [17-21], the mechanism underlying mate recognition remains elusive. *Gryllus firmus* and *G. pennsylvanicus* are morphologically very similar [16], and although calling song is slightly different between species and females from allopatric populations exhibit preference to conspecifics [60], courtship song has not been thought to play a role in mate recognition [61]. In other *Gryllus* species, it did not diverge as much as the calling song [62]. Indeed many female *Gryllus* have preferences for particular calling songs and may use them to identify conspecific males [60,63-66], while courtship song (the only songs females in this study were exposed to) does not seem to be as important. In many gryllids the intensity of courtship behavior (rather than the characteristics of song) is the determining factor in male mating success [67,68]; even muted males can acquire matings after proper courtship behavior [69]. We confirmed that the differential success between conspecific and heterospecific crosses is unlikely to be the result of differences in courtship song, which did not differ between the two species, despite the very high variability among individuals (see Results). This is consistent with previous studies indicating that many variations in courtship song will elicit mating and that the lack of directional selection on courtship song may lead to increased variation in natural populations due to random drift [67-69].

### Cuticular hydrocarbons may mediate mate choice

Because males court conspecific and heterospecific females differentially - *G. firmus* and *G. pennsylvanicus* males court heterospecifics at 58.6% and 48.7% of the rate for conspecifics, respectively (and mate at 35.3% and 70.3%, respectively) - they must be able to differentiate between female species. We suggest that the cuticular hydrocarbon profile might be the phenotypic signal used for mate recognition. In crickets, antennal contact and vibration are essential to the initiation of courtship [41,70], and also ensure detection of the CHC non-volatile compounds. The importance of CHC for male courtship behavior has been demonstrated in *Gryllus bimaculatus*[32,71] where males only exhibited courtship behavior towards females with CHC extracts, and *Teleogryllus oceanicus* which use CHC to identify genetic similarity [33,72] and increase female motivation to reach a calling male [73]. To test the role of CHC compounds in *G. firmus* and *G. pennsylvanicus* mate recognition we conducted preliminary experiments exposing *G. firmus* males to female CHC extracts. Extract on filter disks led to agitated behavior, but no courtship. A few males responded to female cadavers with CHCs intact (2 out of 4) and female cadavers stripped of CHC and re-painted with CHC extract (2 out of 5), but no males courted female cadavers stripped of CHC (n = 5). Together with the CHC differences observed between female species, these preliminary results suggest that CHC might be the mechanism behind the pre-mating behavioral gene exchange barrier.

We observed large differences between male and female CHC profiles within each species (Figure 4); males never exhibited five of the 10 female peaks and most females exhibited more peaks than any male (Figure 3). This sexual dimorphism has also been observed in *G. bimaculatus* and other orthoptera species [33,71] and it is assumed to be a result of sexual selection [74]. Surprisingly, *G. firmus* and *G. pennsylvanicus* males had the same CHC composition, although with differences in peak abundances, while females had differences in both composition and abundance of CHC peaks (albeit with overlap, Figures 3 and 4). It is possible, that males use the CHC profiles to discriminate between conspecific and heterospecific females and adjust their courtship intensity accordingly.

Interestingly some females had CHC composition similar to that of a male (“male-like”, Figures 4 and 5). These females constituted 32.1% of the *G. pennsylvanicus* and 11.6% of the *G. firmus.* To our knowledge, CHC “male like” females (Figure 5) have not yet been described and we currently do not know the behavioral implications of this phenotype. Perhaps it represents an example of male mimicry driven by sexual conflict [75]; more specifically a chemical, rather than morphological or behavioral, male mimicry. Indeed male mimicry (morphological and behavioral) is common in many species of odonata, and has evolved as a sexual harassment avoidance strategy [76-78]. Sexual harassment by males disturbs female energy budgets and may cause physical harm, ultimately reducing fecundity [76,79,80]. In crickets, male harassment is known to reduce female longevity [81], males will also attempt to sequester females into burrows [82,83], mate guard [84,85] and may aggressively prevent spermatophore detachment [86]. It is possible that the excessive polymorphism observed in female CHC profiles may confuse males [87] and reduce excessive male attention by interfering with the mechanisms males use to recognize potential mates, making them unable to effectively adapt to any particular female morph [88] and reducing harassment to the unusual female phenotypes. Furthermore, because this “male-like” phenotype is common to both species, it is possible that most heterospecific crosses happen through these females, we are currently investigating this hypothesis.

## Conclusion

Here we demonstrated that male choice plays a larger role in the pre-mating interspecific behavioral barrier than previously recognized and that the CHC composition might be behind the mate recognition mechanism. Cuticular hydrocarbon profiles are not only different between intraspecific males and females, but differ between females of the two species but not between males. Further studies need to be conducted to understand the implications of the female CHC “male like” phenotype and to confirm that males are indeed capable of differentiating between different female CHC profiles.

## Competing interests

The authors declare that they have no competing interests.

## Authors’ contributions

LSM conceived and designed the experiments, analyzed the data and wrote the manuscript. DPR designed the CHC experiments and conducted the chemical analysis. The undergraduate students ZM, JJ and EH performed the experiments, participated in data analysis and wrote the first draft of parts of the manuscript. ELL participated in the experimental design, data collection and manuscript writing. All authors read, made comments on and approved the final manuscript.

## Supplementary Material

Additional file 1: Figure S1 Principal component analysis of CHC abundances. a) *Gryllus firmus* and b) *G. pennsylvanicus* c) males of both species and d) females of both species, “male-like” females are highlighted in a grey background (these females fall inside the male cloud when shown with males). Colors represent populations: *G firmus*, the beach cricket, is shown in sand colors yellow (Guilford, CT) and orange (Pt Judith, RI) while the inland field cricket, *G. pennsylvanicus*, in is shown in dark green (Ithaca, NY) and light green (Pownal, VT). In a) and b) the “male-like” females are within the male cloud and may not be easy to distinguish. In c) and d) individuals marked with an “*” where used in Figure 3 and in d) the “male-like” female marked with a “+” was used in Figure 5. Click here for file
