# Supplementary figures and images for: Barriers to gene exchange in hybridizing field crickets: the role of male courtship effort and cuticular hydrocarbons
Source: BMC Evol Biol. 2014 Mar 28;14:65. doi: 10.1186/1471-2148-14-65 (PMC4137559; doi:10.1186/1471-2148-14-65)

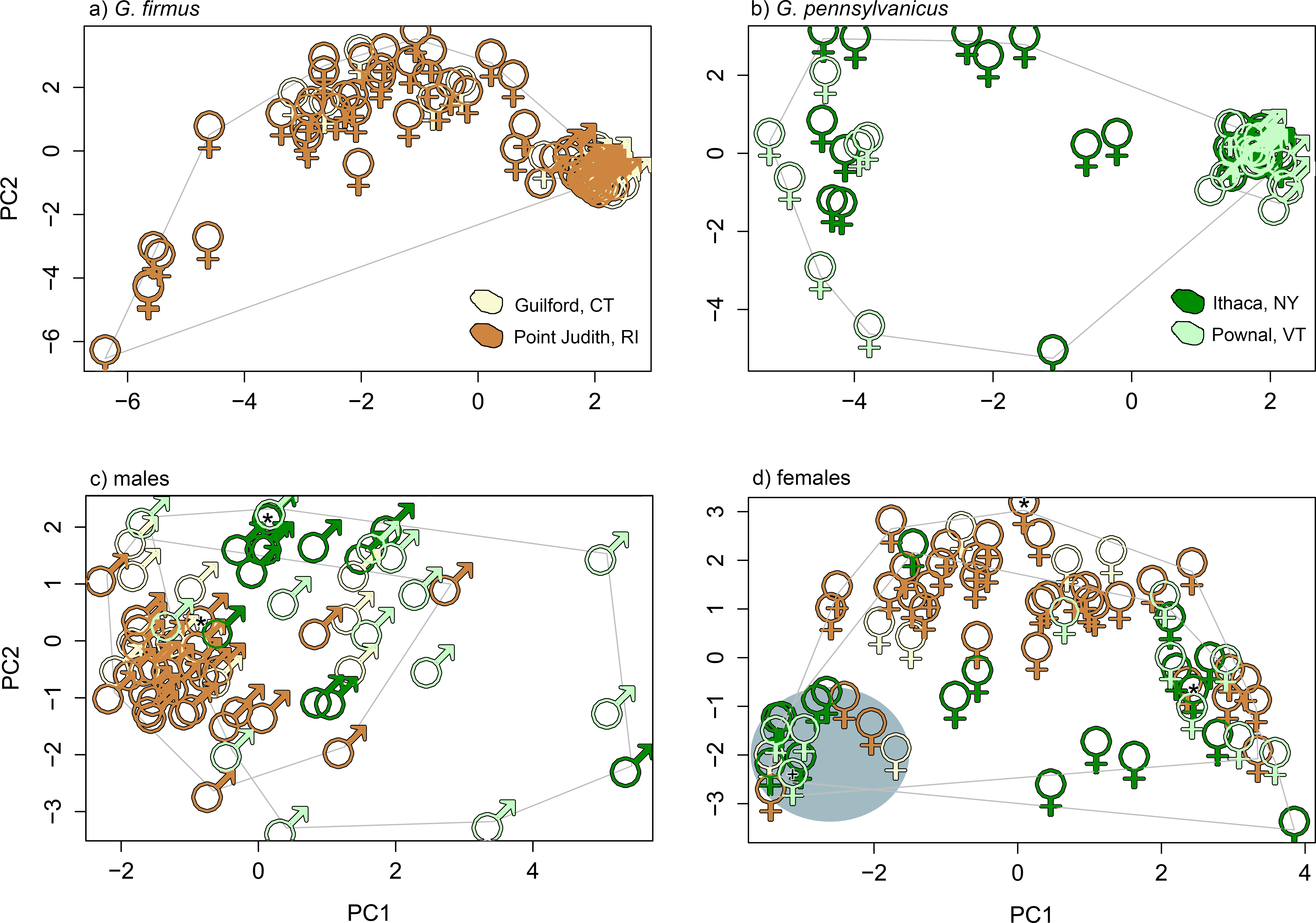

Supplement: Additional file 1: Figure S1 — Principal component analysis of CHC abundances. a) Gryllus firmus and b) G. pennsylvanicus c) males of both species and d) females of both species, “male-like” females are highlighted in a grey background (these females fall inside the male cloud when shown with males). Colors represent populations: G firmus, the beach cricket, is shown in sand colors yellow (Guilford, CT) and orange (Pt Judith, RI) while the inland field cricket, G. pennsylvanicus, in is shown in dark green (Ithaca, NY) and light green (Pownal, VT). In a) and b) the “male-like” females are within the male cloud and may not be easy to distinguish. In c) and d) individuals marked with an “*” where used in Figure 3 and in d) the “male-like” female marked with a “+” was used in Figure 5. [file 1471-2148-14-65-S1.tiff]
